# Intimate partner violence and its associated factors among reproductive-age women in East Africa:-A generalized mixed effect robust poisson regression model

**DOI:** 10.1371/journal.pone.0288917

**Published:** 2023-08-18

**Authors:** Zemenu Tadesse Tessema, Worku Misganaw Gebrie, Getayeneh Antehunegn Tesema, Tesfa Sewunet Alemneh, Achamyeleh Birhanu Teshale, Yigizie Yeshaw, Adugnaw Zeleke Alem, Hiwotie Getaneh Ayalew, Alemneh Mekuriaw Liyew

**Affiliations:** 1 Department of Epidemiology and Biostatistics, Institute of Public Health, College of Medicine and Health Sciences, University of Gondar, Gondar, Ethiopia; 2 Department of Physiology, School of Medicine, College of Medicine and Health Sciences, University of Gondar, Gondar, Ethiopia; 3 Department of Human Anatomy, University of Gondar, College of Medicine and Health Science, School of Medicine, Gondar, Ethiopia; 4 Department of Midwifery, School of Nursing and Midwifery, College of Medicine and Health Sciences, Wollo University, Dessie, Ethiopia; Jawaharlal Nehru University, INDIA

## Abstract

**Background:**

The World Health Organization (WHO) has published estimates revealing that around one out of every three women across the globe has been a victim of either physical and/or sexual violence from an intimate partner or non-partner throughout their lifetime. The available evidence on intimate partner violence in East Africa is limited Consequently, the objective of this study was to evaluate the occurrence and factors linked to intimate partner violence in East Africa.

**Methods:**

The study utilized the most recent data from the Demographic and Health Surveys (DHS) conducted between 2011 and 2018/19 in 11 countries in Eastern Africa. A total of 59,000 women were included in the study. Descriptive and inferential statistics were used to exmine factors associated with IPV. A mixed effect robust Poisson regression model was fitted to identify factors associated with intimate partner violence. The adjusted prevalence ratio (aPR) and its corresponding 95% confidence interval (CI) were employed to determine the presence of a significant association between intimate partner violence and the independent variables.

**Results:**

In this study, the prevalence of intimate partner violence in East Africa was 43.72% with 95% CI 43.32% to 44.12%. In the mixed effect robust Poisson regression model:—Marital status, working status, parity, sex of household headed, wealth index, community poverty, and residence, were significantly associated with intimate partner violence.

**Conclusion:**

The prevalence of intimate partner violence in East Africa is high as compared to the global prevalence 30%, which hinders The Sustainable Development Goals (SDGs), specifically goal 5, aim to attain gender equality and empower women and girls worldwide by the year 2030 Women being previously married and cohabitated, working, having a high number of children, rural residents were positively associated with IPV and household and community wealth index and sex of household headed were negatively related with IPV in East Africa. Therefore, we recommend establishing effective health and legal response using an integrated policy approach and Special attention should be given to women who live rural and poorest to reduce IPV and to achieve Sustainable Development Goals (SDGs) goal 5.

## Background

Intimate partner violence is prevalent among women and encompasses a range of abusive behaviors perpetrated by an intimate partner, such as physical, sexual, emotional abuse, and controlling actions [1]. Violence against women, including intimate partner violence and sexual violence, represents a significant public health issue and a violation of women’s fundamental human rights [2]. According to estimates released by the World Health Organization (WHO), approximately one-third of women worldwide have experienced physical and/or sexual intimate partner violence or non-partner sexual violence at some point in their lives [3]. There is a huge inequalities in IPV 5% in developed countries and 40%. In Sub-Saharan Africa, the prevalence of IPV is 36% which exceeds the global magnitude 30% [3, 4]. Among ever-married or ever-partnered women aged 15 to 49 years in eastern Africa, a notable 38 percent reported experiencing physical or sexual abuse from their intimate partners [5].

Intimate partner violence adversely affects economic and social developments [5]. In addition, The impact of violence on women’s health extends to their physical, mental, sexual, and reproductive well-being, and in certain contexts, it can heighten the risk of HIV transmission [2]. Also, IPV is linked to various mental health concerns, encompassing anxiety, depression, suicidal thoughts and behaviors, as well as posttraumatic stress disorders [6].

Despite, efforts had made to reduce IPV, major public health problem and needs extra effort to alleviate the problem. According to various studies done elsewhere, intimate partner violence is correlated with factor such as age [7–10], residence [7, 9–11], educational status [9, 11–13], income [7–13], sex of household headed [10] exposure to violence in childhood [14], marital status [7, 9], alcohol consumption [8, 15], duration of the relationship [15],substance use [13], social support [13], husband education [12],parity [12].

In spite of diminishing natural resources, inadequate infrastructure such as housing, schools, and health facilities, and rising unemployment rates, the majority of East African countries have yet to establish comprehensive demography and population policies to address intimate partner violence. This form of violence has significant short-term and long-term consequences on women’s physical and mental well-being, and its adverse effects can extend to infants born to women who have experienced IPV. Only 14 East Africa countries had DHS dataset of which 11 of them had DHS dataset between 2011 to 2018/19. As our search concerned there is no multicountry study on IPV using DHS dataset.

To the best of our knowledge, very limited studies conducted so far on intimate partner violence at the East Africa level that include multicountry among reproductive age women (15–49). Previous published artcles used odds ratio(used when cases are rare) to report effect size for this particular study we reported prevalencve ratio(directly compares the prevalence of a condition or outcom) using advanced statistical methods(**robust Poisson regression model**). Therefore, this study was conducted to fill this gap. This study seeks to examine the magnitude and factors associated with intimate partner violence in 11 East Africa countries among reproductive age women (15–49). The results of this study provide valuable insights into the challenges and barriers faced by the WHO East Africa region in achieving the Sustainable Development Goals (SDGs), specifically goal 5, which aims to promote gender equality and empower women and girls by 2030 [16].

## Methods

### Data source, study period, and population

The study utilized data from the most recent Demographic and Health Surveys (DHS) conducted between 2011 and 2018/19 in 11 countries across Eastern Africa.The DHS data sets are globally recognized as nationally representative and comparable surveys, employing standardized manuals, data collection methods, and variables across over 90 countries worldwide [17]. The Demographic and Health Surveys (DHS) collect comprehensive data, encompassing both self-reported and objective measures, in various domains such as reproductive health, fertility, child and maternal health, nutrition, mortality, and self-reported health behaviors among adults [18]. A total of 59,000 reproductive-age women with 98% response rate were included in this study. It includes women who had faced emotional or physical or sexual violence for the last 12 months preceding each country’s survey.

### Measurement of variables

#### Outcome variable

The outcome variables used in this study were derived from a series of questions within the domestic violence module. These questions assessed various forms of violent acts experienced by women. Regarding physical violence, respondents were asked if their last partner had ever engaged in acts such as pushing, throwing objects, slapping, punching, kicking, strangling, burning, threatening with weapons, or twisting arms and pulling hair. Questions on emotional violence focused on whether the partner had ever humiliated, threatened, or insulted the respondent. Sexual violence inquiries involved whether the partner had physically forced the respondent into unwanted sex or other unwanted sexual acts, as well as instances where the respondent had been physically forced to engage in sexual acts she did not want to. Further details on the specific questions for each aspect of intimate partner violence can be found in previous studies

[18]. The survey asked women whether they had experienced any of the specified acts of physical, sexual, or emotional violence from their current or most recent husband/partner in the 12 months prior to the survey. Those who reported experiencing any of these acts were categorized as having experienced intimate partner violence (IPV), while those who did not report any such acts were considered as never having experienced IPV [19].

#### Explanatory variables

Based on known facts and literature [6, 7, 9, 15, 20–23] variables were included in this study. In this study, a range of individual and community-level factors were examined as independent variables. The individual-level factors included the age of respondents (grouped as 15–24, 25–34, and 35+), educational level (categorized as no education, primary, and secondary/higher), husband/partner’s educational level (classified as no education, primary, and secondary/higher), marital status (divided into currently married, cohabiting, and previously married), working status (grouped as not working and working), parity (divided into 0, 1–3, and 4+), and media exposure (categorized as yes or no).

The community variables considered in the study were the place of residence (classified as urban or rural), wealth index (categorized as low, middle, and high), and community-level education (classified as low, middle, and high).

#### Operational definition

*Household wealth index*. The wealth variable used in the analysis was derived from the household wealth index. The dataset categorized households into five quintiles: the lowest quintile (representing the poorest households), the second quintile (indicating poorer households), the third quintile (representing middle-income households), the fourth quintile (indicating richer households), and the fifth quintile (representing the wealthiest households) [18].

*Data analyses*. The 11 East Africa countries DHS data was first downloaded from the dhs program website. Since dhs program uses the same variables name and coding across all countries empirically reviewed variables were kept for analysis. Each country were given a code (Burundi = 1, Comoros = 2, Ethiopia = 3, Kenya = 4, Malawi = 5, Mozambique = 6, Rwanda = 7, Tanzania = 8, Uganda = 9, Zambia = 10, Zimbabwe = 11). The data was appended together to have a single dataset that represent East Africa region. Missing data with respect to the outcome variable were dropped.

To begin with, a descriptive analysis was conducted, utilizing frequency and percentage distributions to examine the characteristics of the participants across each variable outlined in Table 1. Data extraction, cleaning, coding, and analysis were performed using STATA version 14 software (Stata Corp, College Station, Texas, USA). In all analyses, the women population sample weight (v005/1,000,000) was applied to address any over- and under-sampling issues. The "svy" command was used to consider the complex survey design and calculate robust standard errors. Additionally, to assess multicollinearity among the explanatory variables, the variance inflation factor (VIF) was examined, which indicated no evidence of collinearity among the independent variables.To calculate the weighted sample size, non-response and missing data were excluded. The pooled prevalence ratio of intimate partner violence (IPV) was estimated, and a modified Poisson regression model with mixed effects was employed to analyze the relationship between the explanatory variables and IPV. The prevalence ratio and 95%CI were used to measure the effect size. To declare the significant association between intimate partner violence and independent variables confidence interval was used.

**Table 1:** Individual & household/community level characteristics of respondents.

characteristics	Weighted Frequency (N = 59,000)	Weighted Percentage	Women facing intimate partner violence(n = 25,797) 43.73%	p-value
**Intimate partner violence**				
No	33203	56.28	56.28	
Yes	25797	43.72	43.72	
**Age of respondent**				
15–24	14076	23.51	40.05	<0.001
25–34	29487	41.56	44.66	
35 & above	20606	34.93	44.78	
**Women’s level of education**				
No Education	14076	23.86	41.36	<0.001
Primary	29486	49.98	47.68	
Secondary/higher	15437	26.16	38.33	
**Husband/Partner’s level of education (n = 58977)**				
No Education	19570	33.18	44.42	<0.001
Primary	22520	38.18	47.25	
Secondary/higher	16887	28.64	38.26	
**Marital status**				
Currently Married	40133	68.02	40.89	<0.001
Cohabitating	9789	16.59	45.11	
Previously Married	9077	15.39	54.75	
**Working status**				
Working	38478	65.22	47.92	<0.001
Not working	20516	34.78	35.86	
**Parity**				
0	3890	6.65	31.03	<0.001
1–3	31855	54.48	42.65	
4 & above	22725	38.87	47.93	
**Exposure to media**				
Yes	38149	64.66	44.35	<0.001
No	20851	35.34	42.57	
**Sex of household head**				
Male	42568	72.15	43.21	<0.001
Female	16432	27.85	45.06	
**Household/community level**				
**Country**				
Burundi	7647	12.96	50.62	<0.001
Ethiopia	5001	8.48	33.42	
Kenya	4334	7.35	46.79	
Comoros	2538	4.30	10.47	
Malawi	5415	9.18	42.12	
Mozambique	3596	6.10	22.43	
Rwanda	1933	3.28	40.21	
Tanzania	7737	13.11	49.05	
Uganda	7507	12.72	55.60	
Zambia	7372	12.50	46.71	
Zimbabwe	5916	10.03	45.33	
**Place of residence**				
Urban	15431	26.15	41.16	<0.001
Rural	43569	73.85	44.63	
**Wealth index**				
Poorest	12578	21.32	47.30	<0.001
Poorer	11977	20.30	46.31	
Middle	11437	19.39	44.35	
Richer	11908	20.18	43.65	
Richest	11098	18.81	36.31	
**Community education level**				
Low	20313	34.43	43.99	<0.001
Medium	19852	33.65	42.89	
Medium	18834	31.92	44.32	
**Community socioeconomic status**				
Low	19374	32.84	46.89	<0.001
Medium	20306	34.42	43.03	
High	19320	32.75	41.28	

## Results

A total of 59,000 women were included in the study. The largest population of the sample 20,606 (34.93%) of the respondents were age 35 and above, 15,437 (26.16%) of women had secondary education and above, from 59,000 women 16,886(28.64%) of husband/partners had secondary school and above, 40,133 (68.02%) were currently married. A total of 38,149 (64.66%) had mass media exposure at household/community level. High number of study participants 7,737(13.11%) were from Tanzania and the small number 1,933(3.28%) were from Rwanda. A total of 43,569 (73.85%) of women resides in the urban area and 20,306 (34.42%) were from a middle wealth index. A total of 19,852 (33.65%) were from a community with a medium education level, and 19,374 (32.84%) were from a community with low socioeconomic status (**Table 1**).

### Prevalence of intimate partner violence in East Africa among reproductive-age women

Table 1 also presents the prevalence of intimate partner violence among reproductive-age women in East Africa. The prevalence of intimate partner violence was 43.73%. The highest IPV was recorded in Uganda 55.60% and the lowest IPV was recorded in Comoros 10.47%. The IPV prevalence was 47.30% from poorest women and 36.31% from richest women.

### Factors associated with intimate partner violence among reproductive-age women in Eastern Africa

Table 2 displays the adjusted prevalence ratios (PR) and corresponding 95% confidence intervals (CI) that illustrate the relationship between socio-demographic variables and intimate partner violence.The robust Poisson regression model identified marital status, working status, parity, place of residence, wealth index, sex of household head, and community poverty associated with the prevalence of intimate partner violence in Eastern Africa. The finding showed that the prevalence of intimate partner violence among cohabitation and previously married women were 12% (adjusted PR(aPR) = 1.12, 95%CI: 1.09, 1.15) and 53% (aPR = 1.53, 95%CI: 1.48, 1.59) higher prevalence respectively as compared to married women. The prevalence of intimate partner violence among women who had worked were 25% (aPR = 1.25, 95%CI: 1.22, 1.28) higher as compared to women who had no work. Regarding parity primiparous and multiparous women had 34% (aPR = 1.34, 95%CI: 1.28, 1.41) and 48% (aPR = 1.48, 95%CI: 1.40, 1.56) higher prevalence of intimate partner violence respectively, as compared to nulliparous women. The prevalence of intimate partner violence among women who reside in rural is 6% (aPR = 1.06, 95%CI: 1.01, 1.09) higher as compared to women who reside in urban. Wealth index is associated with IPV. As the better wealth index is associated with low prevalence ratio of IPV as compared to women of lowest wealth status as effected size indicated in Table 2. The prevalence of intimate partner violence among household headed women were lower by 10% (aPR = 0.90, 95%CI: 0.87, 0.92) as compared to male household-headed. The prevalence of intimate partner violence among women living communities with in middle and high level of poverty were lower by 5%(aPR = 0.95, 95%CI: 0.92, 0.98) and 7%(aPR = 0.93, 95%CI: 0.90, 0.96) respectively as compared to women in low community poverty.

**Table 2:** Multivariable associations between socio-demographic factors and intimate partner violence in East Africa.

Variables	Intimate partner violence		Prevalence ratio(PR)	
	No	Yes	uPR(95%CI)	aPR(95%CI)
**Age of respondent**				
15–24	8254	5617	1	1
25–34	13571	10951	1.09(1.06,1.12)	1.02(0.99,1.05)
35 & above	11378	9228	1.110(1.07,1.13)	0.98(0.95,1.01)
**Women’s level of education**				
No Education	8255	5820	1	1
Primary	15424	14059	1.17(1.13,1.20)	1.07(0.99,1.10)
Secondary/higher	9520	5919	0.92(0.89,0.95)	1.01(0.97,1.05)
**Husband/Partner’s level of education**				
No Education	10876	8693	1	1
Primary	11879	10640	1.07(1.04,1.09)	1.04(0.98,1.06)
Secondary/higher	10426	6460	0.85(0.83,0.88)	1.06(0.99,1.08)
**Marital status**				
Currently Married	23721	16411	1	1
Cohabitating	5373	4415	1.14(1.11,1.18)	1.12(1.09,1.15)*
Previously Married	4108	4969	1.35(1.32,1.38)	1.53(1.48,1.59)*
**Working status**				
Not working	13158	7357	1	1
Working	20041	18436	1.34(1.31,1.37)	1.25(1.22,1.28)*
**Parity**				
0	2683	1207	1	1
1–3	11267	13587	1.40(1.33,1.47)	1.34(1.28,1.41)*
4 & above	11833	10891	1.56(1.49,1.64)	1.48(1.40,1.56)*
**Exposure to media**				
No	11975	8875	1	1
Yes	21228	16921	1.04(1.02,1.07)	1.02(0.96,1.04)
**Place of residence**				
Urban	9079	6352	1	1
Rural	24124	19444	1.10(1.06,1.13)	1.06(1.01,1.09)*
**Wealth index**				
Poorest	6629	5949	1	
Poorer	6430	5547	1.00(0.97,1.03)	0.96(0.93,0.99)*
Middle	6364	5072	0.95(0.91,0.98)	0.90(0.87,0.93)*
Richer	6710	5198	0.92(0.88,0.95)	0.87(0.84,0.90)*
Richest	7068	4029	0.76(0.73,0.79)	0.74(0.70,0.77)*
**Sex of household head**				
Male	23724	17609	1	1
Female	9314	7390	1.02(1.01,1.06)	0.90(0.87,0.92)*
**Community education level**				
Low	11378	8935	1	1
Medium	11336	8515	0.97(0.93,1.06)	1.00(0.97,1.04)
High	10487	8346	0.98(0.95,1.02)	1.03(0.99,1.06)
**Community socioeconomic status**				
Low	10290	9083	1	1
Medium	11567	8738	0.91(0.88,0.95)	0.95(0.92,0.98)*
High	11345	7974	0.88(0.85,0.91)	0.93(0.90,0.96)*

uPR = unadjusted Prevalence ratio, aPR = adjusted prevalence ratio

## Discussion

The objective of this study is to evaluate the prevalence of intimate partner violence and identify the associated factors among women of reproductive age in East Africa. The study revealed a prevalence of 43.72% for intimate partner violence, with a 95% confidence interval ranging from 43.32% to 44.12%This finding was lower than studies conducted in Uganda 55% [21], and higher studies done in sub-Saharan Africa 37% [24], Saudi Arabia 11.9% [14], Ethiopia 30% [9], DHS multicounty study 34.1% [11], Zimbabwe 40.9% [7], Gambia 40% [25], Benin 15.77% [26]. The difference in the magnitude might be due to the fact that this study was based on the East Africa countries pooled data analysis, which incorporates 11 East Africa countries. In addition, the discrepancy might be the study period difference, and the study population might be another reason. The potential explanation for the lower prevalence found in this study compared to previous studies conducted in Uganda could be attributed to the specific context of the study location. This study was conducted at two health clinics in northern Uganda, specifically in Gulu and Omoro districts, where the local population had experienced significant impacts from the conflict. The possible reason why lower studies sub-Saharan Africa is that the East Africa region faced different conflicts may result this variation.

In the mixed effect modified Poisson regression model, variables such as marital status, working status, parity, residence, sex of household headed, wealth index, and community poverty were significant variables associated with intimate partner violence among reproductive-age women in East Africa. Women who are living with cohabitation and previously married were associated with IPV. The findings of this study align with previous research, which has consistently shown that separated and divorced women have a significantly higher likelihood of experiencing intimate partner violence. Specifically, the likelihood for separated women was reported to be 30 times higher, while for divorced women, it was found to be 9 times higher compared to married women [27]. The possible might be women who are continuing their relationship with as cohabitating and previously married might be following equality principle with their partner for their expense whereas married women may enjoy a shared family income for their expense [28].

This study revealed that women working status is associated with IPV in East Africa among reproductive age women. This finding is consistent with other previous studies done elsewhere which indicated that women are engaged in any form of employment, the incidence of spousal abuse increases by 9.4% [29]. The possible reason might be if women pass their time working there may not have time for their partner and this might increase intimate partner violence against women. However, this finding contradicts studies done in Nigeria which reveals that women who was employed reduced the intimate partner violence than unemployed [30]. The discrepancy might be for our study if women engaged with work she may not have much time for her partner this may result either types of violence. For Nigeria study if a women had no work she may dependent to here partner and economic dependency may be one cause of violence. If a women had her own income there may not be any fight between couples related to expenditure.

This study finding showed parity is associated with IPV in East Africa among reproductive age women. This finding is consistent with studies conducted elsewhere [31]. The possible reason might be the presence of the child may necessitate ongoing partner contact and if the number of children in the family increase the expenditure of the household increases including educational payment for students. In addition, women may not want to leave their matrimonial homes as they may tend to secure the welfare of their children [7].

Residence is associated with IPV. This finding is consistent with studies done in Bangladesh [10], Ethiopia [11, 32, 33], Kenya [34]. The possible reason might be urban areas are significantly less compliant to domestic violence since had a better educational level autonomous, and partners better educational level. However, this finding contradicts other previous studies conducted in Zimbabwe [7], which revealed that being a rural residence decreases the prevalence of intimate partner violence than urban women. The discrepancy might be sample size difference, population difference, and study period.

The sex of the household head is significantly associated with intimate partner violence. The possible reason might be if women are leading the household no one violates their right. This finding is supported by studies conducted in Tanzania [35]. However, some studies had observed the opposite scenario example studies done in Bangladesh being male household-headed decreases intimate partner violence [10], Iran [36]. The discrepancy might be the type of study design like the Iran article done using qualitative study while this study used cross-sectional study.

Individual-level income and community poverty were significantly associated with intimate partner violence. As the wealth status of a woman increases the prevalence of intimate partner violence decreases. This finding is consistent with studies conducted in Tanzania [35], Zimbabwe [31], Bangladesh [12], Myanmar [37], Ethiopia [6]. The possible reason might be women with better wealth status had fewer resource-related disputes and this leads to decreased intimate partner violence.

### Strength and limitation of the study

Our study exhibits both strengths and limitations. One notable advantage is the use of nationally representative datasets from 11 East African countries, allowing for the generalization of findings within the region. However, caution is necessary when comparing results from different surveys due to variations in data collection periods. Another strength is the substantial sample size of 59,000 participants. Nevertheless, the study is vulnerable to social desirability and recall bias as it relies on self-reported data. The study period, spanning from 2011 to 2018/19, may affect the applicability of the findings. Additionally, the surveys being cross-sectional in nature only enable the identification of associations, not causal relationships. For future research, we recommend collecting primary data and adopting case-control study designs to address the challenges of establishing causal relationships.

## Conclusion

The prevalence of intimate partner violence in East Africa is high as compared to the global prevalence 30%, which hinders the Sustainable Development Goals (SDGs), particularly goal 5, which seeks to achieve gender equality and empower all women and girls by 2030. Women being previously married and cohabitated, working, having a high number of children, rural residents were positively associated with IPV and household and community wealth index and sex of household headed were negatively related with IPV in East Africa. Therefore, we recommend establishing effective health and legal response using an integrated policy approach and Special attention should be given to women who live rural and poorest to reduce IPV and to achieve Sustainable Development Goals (SDGs) goal 5. For furure researcher we encourage to collect primary data and case control study deign to solve cause effect dilemma.

## Abbreviations

aPR: adjusted Prevalence ratio
CI: Confidence Interval
DHS: Demographic and Health Survey
IPV: Intimate Partner Violence
SDG: Sustainable Development Goa
uPR: unadjusted Prevalence Ratio
VIF: Variance Inflation Factor
WHO: World Health Organization
